# Medical Students’ Perception Regarding Health Policy Teaching and Their Participation in Health Policy Roles: A Survey at a Public University in Malaysia

**DOI:** 10.3390/healthcare10060967

**Published:** 2022-05-24

**Authors:** Mainul Haque, Nor Azlina A Rahman, Sayeeda Rahman, Md. Anwarul Azim Majumder, Sharifah Shasha Binti Syed Mohdhar, Halyna Lugova, Adnan Abdullah, Shahidah Leong Binti Abdullah, Mohd Hafizi Bin Ismail, Jaykaran Charan, Santosh Kumar, Mohammed Irfan, Ibrahim Haruna Sani, Abdullahi Rabiu Abubakar, Kona Chowdhury, Farhana Akter, Dilshad Jahan, Rahnuma Ahmad

**Affiliations:** 1 Unit of Pharmacology, Faculty of Medicine and Defence Health, Universiti Pertahanan Nasional Malaysia (National Defence University of Malaysia), Kem Perdana Sungai Besi, Kuala Lumpur 57000, Malaysia; 2 Kulliyyah of Allied Health Sciences, International Islamic University Malaysia, Kuantan 25200, Malaysia; nazara@iiumn.edu.my; 3 School of Medicine, American University of Integrative Sciences, Bridgetown BB11114, Barbados; srahman@auis.edu; 4 Faculty of Medical Sciences, The University of the West Indies, Cave Hill Campus, Bridgetown BB11000, Barbados; azim.majumder@cavehill.uwi.edu; 5 Unit of Internal Medicine, Faculty of Medicine and Defence Health, Universiti Pertahanan Nasional Malaysia (National Defence University of Malaysia), Kem Perdana Sungai Besi, Kuala Lumpur 57000, Malaysia; shashamohdhar@gmail.com; 6 Humanitarian Assistance and Disaster Relief Centre, Faculty of Medicine and Defence Health, National Defence University of Malaysia, Sungai Besi Prime Camp, Kuala Lumpur 57000, Malaysia; halina@upnm.edu.my; 7 Unit of Community Medicine, Faculty of Medicine and Defence Health, National Defence University of Malaysia, Sungai Besi Prime Camp, Kuala Lumpur 57000, Malaysia; 8 Unit of Occupational Medicine, Faculty of Medicine and Defence Health, Universiti Pertahanan Nasional Malaysia (National Defence University of Malaysia), Kuala Lumpur 57000, Malaysia; dradnanabdullah@upnm.edu.my; 9 Unit of Military Medicine, Clinical Psychologist, Faculty of Medicine and Defence Health, Universiti Pertahanan Nasional Malaysia (National Defence University of Malaysia), Kem Perdana Sungai Besi, Kuala Lumpur 57000, Malaysia; shahidah2013@gmail.com; 10 Unit of Administration, Faculty of Medicine and Defence Health, Universiti Pertahanan Nasional Malaysia (National Defence University of Malaysia), Kem Perdana Sungai Besi, Kuala Lumpur 57000, Malaysia; hafizi@upnm.edu.my; 11 Department of Pharmacology, All India Institute of Medical Sciences, Marudhar Industrial Area, 2nd Phase, M.I.A. 1st Phase, Basni, Jodhpur 342005, Rajasthan, India; dr.jaykaran78@gmail.com; 12 Department of Periodontology and Implantology, Karnavati School of Dentistry, Karnavati University, 907/A, Uvarsad, Gandhinagar 382422, Gujarat, India; drsantoshkumar2004@gmail.com; 13 Instituto Odontologico das Américas (IOA-Pelotas), 1399—Centro, Pelotas 96020-360, RS, Brazil; irfan_dentart@yahoo.com; 14 Unit of Pharmacology, College of Health Sciences, Yusuf Maitama Sule University (YUMSUK), Kofar Kansakali-700282, Kano PMB 3220, Nigeria; harunaibrahim81@yahoo.com; 15 Department of Pharmacology and Therapeutics, Faculty of Pharmaceutical Sciences, Bayero University, Kano-700233, Kano PMB 3452, Nigeria; unisza7@gmail.com; 16 Department of Paediatrics, Gonoshasthaya Samaj Vittik Medical College and Hospital, Dhaka 1344, Bangladesh; konaonu96@gmail.com; 17 Department of Endocrinology, Chittagong Medical College, Chattogram 4203, Bangladesh; fakter36@gmail.com; 18 Department of Hematology, Asgar Ali Hospital, 111/1/A Distillery Road, Gandaria Beside Dhupkhola, Dhaka 1204, Bangladesh; dr.dilshad.bd@gmail.com; 19 Department of Physiology, Medical College for Women and Hospital, Dhaka 1230, Bangladesh; rahnuma.ahmad@gmail.com

**Keywords:** health, policy, knowledge, perception, medical students, Malaysia

## Abstract

Background: Health policy is a set of comprehensive principles and legislations that guide how healthcare should be effectively delivered in the community. Medical schools should prepare students to undertake managerial responsibilities by incorporating health policy into the curriculum to deal with the intricacies of healthcare systems and their clinical roles in their future professional careers. Objective: To examine medical students’ perception at a Public University in Malaysia regarding teaching health policy and their participation in health policy roles. Material and Methods: A cross-sectional study using universal sampling was carried out among the medical students using a paper-based questionnaire to collect the data. Results: Most respondents opined their willingness to learn health policy (80.9%) and that teaching health policy (83.6%) should be compulsory for medical students. The respondents thought health policy should be introduced earlier in Year 1 or 2. The student scores on their knowledge regarding health policy and year of study were significantly associated with their involvement in the health policy roles in both the simple and multiple logistic regression. Both statistical tests reported higher participation in health policy roles with the higher year of study, though only Year 4 and 5 were significant in the simple logistic regression and only Year 5 in the multiple logistic regression compared to Year 1. On the other hand, age and type of admission show significant results only in the simple logistic regression, while the race was only significant at the multivariate level. Conclusions: This study demonstrated that most respondents showed their willingness to learn health policy, participate in the health policy programs, and recommend that health policy be considered an essential topic in the medical curriculum, which should be taught right from the first year of medical school. We recommend encouraging students’ participation in health policy activities.

## 1. Introduction

The World Health Organization (WHO) defines health policy as the verdicts, strategies, and activities to accomplish precise health care goals within the social order [1,2]. Additionally, health policy has been defined as predictive and involves short- and medium-term targets and milestones for effective health care [3]. Health policy usually attempts to improve population health [4], and healthcare policy goals are typically created to provide equitable and efficient access to high-quality healthcare services [4,5]. A well-planned health policy could help mobilize adequate resources to a priority healthcare program to ensure effective distribution of limited resources from tertiary healthcare facilities to the primary healthcare facilities located in the communities. It is necessary to include healthcare professionals in health policy formulation and decision-making of effective allocation of resources [6,7,8,9,10].

Multiple reports mentioned that several developments and civilization goals were actualized through effective health policy formulation [11,12]. In developing countries with scarce resources and diverse healthcare needs, health policy is crucial in determining equitable, efficient, and effective healthcare services [13,14]. Effective health policy is a yardstick for predicting and realizing future health needs, such as personnel, volunteers, infrastructures, and community integration to seasonal diseases and global pandemics [15,16,17]. Furthermore, understanding international health policies is becoming very important in the modern world due to the emergence of a worldwide pandemic like COVID-19 [18,19,20].

Health policy including healthcare management and health economics are considered essential components of the training curricula for all categories of healthcare professional students [21,22,23,24]. It has been highlighted by many studies and reports that health professionals should have a sound knowledge of all aspects of the fields, as mentioned earlier [25,26,27]. Mou et al. [28] greatly emphasized that: “In order to provide comprehensive care for patients and effectively participate in health care reform, the medical community must be literate in health policy. … health policy literacy should no longer be considered an ancillary skill, but rather a core competency of a 21st-century physician”. To build a solid foundation for effective health policies, the health professional students should be educated in these fields, including knowledge of medicine, pharmaceuticals, behavioral sciences, biomedical, and environmental sciences [3,26,29,30,31,32,33]. Despite the tremendous significance of learning health policies among health professionals, especially medical doctors globally, in determining the success of the prevention of diseases [26,34,35], many medical schools around the globe yet not incorporated health policies issues into their curricula adequately [7,9,11,36,37,38,39]. Multiple studies highlighted the lack of standardized guidelines to develop and implement an effective health policy curriculum necessary for medical graduates to practice medicine [40,41,42]. According to a study conducted in the USA by Patel et al. [43], nearly half of graduating medical students reported that they received inadequate teaching in topics related to health policy. Another study conducted among medical students in the USA found that 54% expressed dissatisfaction with the health policy teaching, though 96% of respondents felt that health policy knowledge is important for their future career [44]. The study surveyed medical students in Ontario and California and found that the teaching of health care policy was judged inadequate by 73.1% and 57.5% of students in California and Ontario, respectively [10]. Mou et al. [28] surveyed the deans of medical education (*n* = 93) in the United States and found that “there is room for expansion of health policy education within the curriculum: 58% of respondents reported that their school currently has ‘too little’ health policy education”. Consequently, incorporating population-based health policy and guidelines into the medical curriculum is a longstanding priority [7,9,11,27,38,39,40,41].

The Institute of Medicine (IOM), USA has identified the gap and recommended the health policy training for public health professionals: “Although the importance of policy has long been recognized, education in policy at many schools of public health is currently minimal. Education in policy analysis, policy development, and the application of policy must be addressed” [29]. Dempsey et al. (2011) in their CompHP Core Competencies Framework for Health Promotion Handbook clearly highlighted the importance of the teaching and training of health policy: “A competent workforce that has the necessary knowledge, skills and abilities in translating policy, theory and research into effective action is recognised as being critical to the future growth and development of global health promotion” [45]. A review of the literature identified the lack of an effective method to teach health policy or delivery of national healthcare programs within the current format of undergraduate medical curriculum delivery [28,39,43,45,46]. Patel et al. [46] highlighted barriers which medical schools encountered in relation to health policy teaching: overcrowding of curriculum with scientific and clinical information, lack of an interdisciplinary faculty team to teach health policy, and limited evidence-based research on current innovative teaching methods of health policy. Another study conducted in the USA identified curricular inflexibility, lack of faculty and student interest, and limited prerequisite and financial resources as the important constraints on integrating health policy into medical curriculum [28]. A well-designed public health curriculum may help students see the value and importance of this field as they move through medical school and prepare to enter professional practice [10,28,44,47,48]. Moreover, the importance of evaluating and remodeling the current health policy curricula, and, particularly, adopting a new “service learning” approach to facilitate student involvement in health policy, can never be overemphasized [47]. Evidence from some medical schools suggested that training in health policy can be successfully integrated into medical school curricula with positive outcomes in relation to students’ self-reported knowledge and confidence [40,41,49,50,51,52,53]. This research aimed to evaluate medical students’ perception of a Public University in Malaysia regarding teaching health policy and their participation in health policy roles. The outcome would help the policymakers improve medical students’ curricula, improve their practice after graduation, and prepare them for future global health challenges. 

## 2. Materials and Methods

### 2.1. Study Design

This study used a cross-sectional study to collect the data from medical students at a Public University in Malaysia.

### 2.2. Study Population and Sampling Method

The study population was both the preclinical and clinical years’ medical students from years 1 to 5 in a Public University in Malaysia. Year 1 to Year 5 students of both genders and of all ages from Malay, China, and India were included in the study. The survey was conducted using a universal sampling method.

### 2.3. Study Period

Data collection was carried out from 14 January to 28 March 2019 using the self-administered questionnaire. The students were given the hard copy of the questionnaire at a prearranged free time in the lecture hall, and they answered the questionnaire on their own.

### 2.4. Data Collection Tool (Questionnaire)

A validated questionnaire about health policy was adopted from an earlier study conducted by Malik et al., 2017 [39]. Hence, the same question was used for the outcome measure with yes and no answers. It might not be adequate, as pointed out, but the validation study conducted was quite good, and hence we feel that it was adequate for this study too. We have planned to conduct another study in the upcoming days to address the inadequacy of well-defined variables. The study instrument was again pretested and validated in the local context. The questionnaire was administered to 15 (3 × 5 = 15) medical students who did not participate in the principal study. Their responses were collected and analyzed for validity and reliability. The Cronbach alpha was calculated as 0.73. Our value was within the passable alpha range, from 0.70 to 0.95 [54], and determined an adequate measure of reliability or internal consistency of our instrument. A total of 238 (253-15 non-participants) questionnaires were given to the study respondents after a prearranged lecture class. The sociodemographic variables collected using this questionnaire were age in years, gender (male and female), race (Malay, Chinese, Indian), year of study (year 1 to 5), type of admission (cadet officer, territorial army, civil servants), and whether they had any family member currently working in the health-related field (yes/no).

The outcome variable in this study was the earlier or current involvement of the medical students in any health policy role, which was coded as ‘Yes’ and ‘No’. A few other questions on health policy were also coded as ‘Yes’ and ‘No,’ namely whether they would like to be taught about health policy and whether they thought medical students should receive compulsory teaching on health policy as part of the curriculum. Similarly coded were the factors which the students perceived as the factors which prevent them from being involved in the student health policy committee in the university, namely lack of time, lack of knowledge, no interest in health policy, lack of awareness of the available opportunities, a belief that students cannot impact health policy, or others. However, no elaborations were given on the other factors mentioned. Another question asked to determine in which year of study the respondent thinks the teaching on health policy should be introduced in medical school (Year 1 to 5) and they were also asked to score how much they know about health policy from 1 (nothing) to 5 (very knowledgeable).

### 2.5. Ethical Consideration 

This research was approved by the Institutional Research Ethics Committee from the Centre for Research, Innovation, and Management, National Defense University, Malaysia [Code of Research: SF0043-UPNM/2018/SF/SKK/06, Memo No: UPNM (PPPI) 16.01/06 Jld 2 (21), 6 June 2018]. An information sheet was given to each student to read, and they would provide their written consent on a consent form before the data collection was conducted. The students were allowed to withdraw, and their privacy and confidentiality were always guarded.

### 2.6. Data Analysis 

Data analysis was done using STATA Intercooled version 15.1 software (StataCorp, 4905 Lakeway Dr, College Station, TX 77845, USA). Descriptive statistics were reported in the form of frequency and percentages for the categorical variables and mean and standard deviation (SD) for the numerical variable. 

Binary logistic regression was used to evaluate the factors associated with the students’ involvement in health policy. The univariate analysis was conducted on all the possible factors using simple logistic regression, followed by multiple logistic regression. Manual forward and backward regression were carried out by initially fitting all the variables in the model and removing any non-significant variables one by one. Then, the variables were put into the model again, one by one, and removed if they were still not significant. The procedure was carried out until the final model was fit with only all the significant variables included. In the classification table, Ifit (Pearson Chi2 goodness-of-fit test), lfit group (10) (Hosmer-Lemeshow Chi2 goodness-of-fit test), lsens (sensitivity and specificity graph), lroc (area under the ROC curve), and VIF (variation inflation factor) were mentioned to highlight that the model fitness was checked for the logistic regression done and was found adequate to support the validity of the statistical analysis done. They were not the main results under focus, which was why those results were not elaborated in the manuscript.

Furthermore, we had conducted the multiple logistic regression analysis. The Pearson and Hosmer-Lemeshow Chi2 goodness-of-fit test showed the *p*-values of 0.224 and 0.162, respectively, which are more than the significance level of 0.05, indicating that the model was fit. The variation inflation factor for the numerical variable was 1.0, which is less than 10 (or the stricter cut-off of 2.5), indicating that there is no multicollinearity problem in the model. The variables were correctly classified in the classification table and the area under the ROC (Receiver Operating Curve) was 70%, which, even if not great, shows that the results are fair. In conclusion, the checking showed that the model fitness of the multiple logistic regression done was good.

However, the pseudo-R-square was quite low with the value of 0.176, meaning the variables used in this study only explained a small portion of the involvement of the students with the health policy roles. Further study is needed to explore the other factors to better explain the situation. On the other hand, the software does not give the regression coefficient values for the results of the multiple logistic regression done; instead, it already converted the regression coefficient for each independent variable into an odds ratio for the results presentation. The significance level was set at 0.05 for the 95% confidence interval.

## 3. Results 

Out of 238, 209 who responded to the questionnaire, and therefore the response rate was 87.81%. The sociodemographic information is shown in Table 1. The mean age of the students was 21.5 ± 1.11 years. The majority of the participants were male (50.7%), Malay (74.2%), and 71.6% did not have any family member working in a health-related field. According to the type of admission, the highest participation was from territorial army (19.2%) students. More than a quarter of the medical students (27.4%) were involved in health policy roles during the data collection.

The response related to teaching and knowledge of health policy is summarized in Table 2. It is reassuring to know that a majority of the students (80.9%) would like to be taught about health policy and recommended that it be made compulsory (83.6%). More than one-third of the students (34.1%) opined that health policy should be introduced earlier in Year 1 of the curriculum. More than half of the students scored 3 (Scale: 1-nothing to 5-very knowledgeable) when asked about knowledge of health policy.

The most common factors preventing the students from being involved in the student health policy committee were lack of time (47.4%), followed by unawareness of the available opportunities (40.2%) and lack of knowledge (39.2%) (Table 3).

Table 4 and Table 5 show the simple and multiple binary logistic regression results, respectively, in assessing the factors associated with the student’s involvement in health policy roles. As shown in Table 4, age, scores on knowledge about health policy, years 4 and 5, and the territorial army were found to be significantly associated with the student’s involvement in health policy roles at the univariate level. The simple logistic regression shows 1.5 times higher odds of getting involved in a health policy role with every one-year increment in age. Similarly, for every unit increment in the scoring on knowledge about health policy, there were 2.7 times higher odds of getting involved in a health policy role. Year 4 and Year 5 students were also found to have higher odds (1.7 and 3.7 times, respectively) than Year 1 students of getting involved in a health policy role. However, students admitted to the territorial army had about three times lower odds of getting involved in a health policy role than those admitted as cadet officers.

On the other hand, Table 5 shows the final model of the multiple logistic regression with scores on knowledge about health policy, being Indian, and year 5 were significantly associated with the student’s involvement in health policy roles at the final multivariable level. Similar to the simple logistic regression result, multiple logistic regression also found that there were 2.7 times higher odds of getting involved in a health policy role for every unit increment in the score on knowledge about health policy. It was also noted that the odds of getting involved in health policy roles increased with each year of study. However, on this multivariate level, a significant association was only found between Year 1 and Year 5. The Year 5 students were 4.3 times at higher odds of being involved in the health policy roles than the Year 1 students. Ethnicity was another variable that was significantly associated with the student’s involvement in health policy roles at the multivariate level. Indian students were found to be least involved in health policy roles, and were 0.2 times as likely to be involved compared to the Malay students.

## 4. Discussion

In the present study, more than four-fifths of the respondents showed their willingness to learn health policy (81%) and mentioned that teaching health policy should be compulsory for medical students (83%). Several studies proposed similar recommendations to include these aspects in medical curricula [9,10,28,39,40,41,46]. More than one-third (34.5%) of the respondents recommended that health policy be taught right from the first year of medical school. The study conducted by Malik et al. found that 77% of students in the UK medical schools expressed their willingness to be taught health policy [39]. In comparison, 73% recommended compulsory teaching of health policy at undergraduate levels. The knowledge level of our students (3 out of 5) was higher than the knowledge of the UK students (2/5). The study conducted in three USA medical schools found that students felt less strongly (2.85/5) about their knowledge regarding health policy-related current events [50]. Our study also showed 2.7 times higher odds of getting involved in a health policy role for every unit increment in the score on knowledge about health policy. A new curriculum on health care policies and systems at the Keck School of Medicine of the University of the South Carolina (USA) demonstrated significantly higher post-curriculum scores (16%) among first-year medical students [49]. Another study showed that a four-week online health policy elective for medical students during the COVID-19 pandemic increased their knowledge and skills relating to essential health policy topics [40].

There is a growing recognition of the need to develop a need-based medical curriculum to train tomorrow’s doctors to address health systems changes and challenges healthcare professionals face in their professional lives [51]. Various reports and studies highlighted the role of health policy and health economics in training medical students to prepare them for future professional positions [50,51,53,55,56,57,58]. A considerable mismatch was observed in teaching health policy and health economics across the medical schools about content coverage, time allocation, and delivery contents [53,59]. Many studies highlighted the absence of knowledge and application of health policy principles and health economics in medical education and clinical practice [28,55,58,60,61,62,63]. Better student knowledge was observed among medical students who received teaching by health economists using a structured curriculum [53,59]. However, healthcare management has attracted considerable attention from medical educationists in recent years, and there is evidence that these aspects have been accommodated in the overcrowded health professional curricula. The ongoing COVID-19 pandemic has emphasized “the study of healthcare, health efficacy, health policy, and health research” and pushed these agendas to “the front of the academic line” in dealing with “allocation of healthcare resources, payment for health services, and research and development of vaccines” [61]. Health policy reform is a global agenda, and the COVID-19 pandemic emphasized the “need for political advocacy from the medical community” [40].

The present study also found that more than one quarter (27%) of the medical students positively opined on their involvement in the health policy activities, which is higher than the finding (6%) reported by Malik et al. (2017) [39]. We also found that the odds of getting involved in health policy roles increased by year of study—Year 4 and Year 5 students have a higher chance (1.7 and 3.7 times, respectively) of getting involved than Year 1 students. Several research findings revealed that medical students were interested in participating in health policy projects [10,39]. Other studies mentioned that students and faculty members were interested in participating in training related to health policy and related competencies [44,62,63]. Medical students in our study suggested that they could help in the health policy-making process by direct participation or volunteering; however, lack of time is a limiting factor when it comes to participating in health policy activities, which medical students in the UK also highlighted [39]. A US medical school study showed that 94% of schools offer health policy education but no structured program guidelines to teach such courses [28]. During the medical admission interview, prospective applicants were questioned about the current state and challenges of the healthcare system, healthcare reforms, and proposed solutions to improve medical care [64].

Teaching health policy at undergraduate levels is a real challenge, especially adding health policy topics into an already crowded curriculum “would put undue strain on both the medical school curricula and the students” [38]. Though various authorities have highlighted the importance of health policy, the medical education system failed to adequately prepare future health professionals for the critical public health challenges [10]. Concerns have been expressed that tomorrow’s doctors will face significant challenges in providing evidence-based care because of a lack of proper training in fundamental concepts of health policy [57,58,65]. It has been recommended that integrating health policy topics in the student and resident curricula will go a long way to improve healthcare service delivery at the community level [43,56,57,58].

## 5. Limitation of the Study

This cross-sectional study involved only medical school and had a small sample size; therefore, caution should be taken to generalizing the data to other settings.

## 6. Conclusions and Recommendations

The present study demonstrated that the majority of the respondents showed their willingness to learn health policy and recommended that health policy be considered an essential topic in the medical curriculum, which should be taught right from the first year of medical school. The majority of respondents were interested in participating in the health policy programs, but lack of time is the main hindrance. Future doctors need to participate in health policy decision-making during their training. The best solution is to incorporate health policy into the medical student’s curriculum, and the teaching should begin from year 1 of medical school. We also recommend encouraging students’ participation in health policy activities and allowing students to take part in health policy-making activities.

## 7. Article Highlights

1. Health policy is a set of comprehensive principles and legislations that guide how healthcare should be effectively delivered in the community.
2. Healthcare professionals should be involved in health policy formulation and decision-making to effectively allocate resources to improve population health.
3. A curriculum that includes health policy can help students comprehend the significance of this field and learn to implement this knowledge in their future medical practice.
4. Incorporating health policy into the medical curriculum from the early years of medical school would help develop public health-oriented physicians.

## Figures and Tables

**Table 1:** Sociodemographic information of the medical students (*n* = 209).

Variable	Frequency	Percentage
Gender (*n* = 209):		
Male	106	50.7
Female	103	49.3
Race (*n* = 209) ^#^		
Malay	155	74.2
Chinese	16	7.7
Indian	38	18.2
Year of study (*n* = 209) ^#^		
1	50	23.9
2	45	21.5
3	59	28.2
4	38	18.2
5	17	8.1
Type of admission (*n* = 208) *		
Cadet officer	72	34.6
Territorial army	40	19.2
Civil servants	96	46.2
Any family member works in the health-related field (*n* = 208) *		
Yes	59	28.4
No	149	71.6
Earlier or current involvement in any health policy role (*n* = 208)		
Yes	57	27.4%
No	151	72.6%

^#^ Total percentage is not 100% due to the rounding-up. * With one missing value.

**Table 2:** Responses to health policy teaching and knowledge (*n* = 209).

Variable	Frequency	Percentage
Would like to be taught about health policy (*n* = 209):		
Yes	169	80.9
No	40	19.1
Thought medical students should receive a compulsory teaching on health policy (*n* = 208) *:		
Yes	174	83.6
No	34	16.4
Year of study, they think health policy should be introduced in medical school (*n* = 164) *^#^:		
1	56	34.1
2	27	16.5
3	45	27.4
4	15	9.1
5	21	12.8
Scoring on how much they know about health policy (*n* = 209):		
1 (nothing)	16	7.7
2	28	13.4
3	110	52.6
4	48	23.0
5 (very knowledgeable)	7	3.3

^#^ Total percentage is not 100% due to the rounding-up.* With some missing value.

**Table 3:** The perceived factors preventing the students from participating in the health policy committee (*n* = 209).

Variable	Frequency	Percentage *
Lack of time	99	47.4
Lack of knowledge	82	39.2
No interest in health policy	34	16.3
Unaware of available opportunities	84	40.2
Believe that students cannot impact health policy	21	10.0
Others	19	9.1

* The total 100% is for each factor as students can choose more than one answer.

**Table 4:** Simple logistic regression of factors associated with the student’s involvement in health policy roles (*n* = 209).

Variable	Odds Ratio (95% CI)	*p*-Value
Age	1.501 (1.039, 2.168)	**0.030**
Scores on knowledge about health policy	2.663 (1.716, 4.132)	**<0.00**1
Gender:		
Male	1.000	-
Female	0.978 (0.531, 1.800)	0.944
Race:		
Malay	1.000	-
Chinese	2.581 (0.911, 7.313)	0.074
Indian	0.484 (0.189, 1.240)	0.130
Year of study:		
1	1.000	-
2	0.829 (0.264, 2.607)	0.748
3	1.187 (0.981, 6.337)	0.055
4	1.742 (1.263, 9.283)	**0.016**
5	3.664 (1.751, 19.926)	**0.004**
Type of admission:		
Cadet officer	1.000	-
Territorial army	0.353 (0.130, 0.956)	**0.041**
Civil servants	0.794 (0.409, 1.540)	0.495
Any family member works in the health-related field:		
No	1.000	-
Yes	1.784 (0.928, 3.432)	0.083
Would like to be taught about health policy		
No	1.000	-
Yes	0.994 (0.459, 2.152)	0.988
Thought medical students should receive compulsory teaching on health policy:		
No	1.000	-
Yes	1.897 (0.740, 4.861)	0.182
Year of study, students think health policy should be introduced in medical school:		
1	1.000	-
2	1.227 (0.398, 3.782)	0.721
3	1.848 (0.742, 4.602)	0.187
4	1.023 (0.246, 4.259)	0.975
5	1.636 (0.516, 5.187)	0.403
Perceived factors preventing the students from being involved in the student health policy committee:		
Lack of time:		
No	1.000	-
Yes	0.737 (0.398, 1.363)	0.331
Lack of knowledge:		
No	1.000	-
Yes	0.698 (0.369, 1.323)	0.271
No interest in health policy:		
No	1.000	-
Yes	0.945 (0.412, 2.170)	0.894
Unaware of available opportunities:		
No	1.000	-
Yes	1.218 (0.657, 2.258)	0.531
Believe that students cannot impact health policy:		
No	1.000	-
Yes	1.486 (0.561, 3.937)	0.425
Others:		
No	1.000	-
Yes	1.622 (0.605, 4.349)	0.337

CI = confidence interval. Bold = significant categories.

**Table 5:** Multiple logistic regression of factors associated with the student’s involvement in health policy roles (*n* = 209).

Variable	Odds ratio (95% CI)	*p*-Value
Scores on knowledge about health policy	2.663 (1.614, 4.297)	**<0.00**1
Race:		
Malay	1.000	-
Chinese	1.328 (0.410, 4.303)	0.637
Indian	0.222 (0.072, 0.685)	**0.009**
Year of study:		
1	1.000	-
2	0.572 (0.171, 1.910)	0.364
3	1.843 (0.683, 4.973)	0.228
4	2.265 (0.711, 7.214)	0.166
5	4.347 (1.138, 16.609)	**0.032**

CI = confidence interval; Bold = significant categories.

## Data Availability

The datasets of the current study are available from the corresponding author on reasonable request.
